# Association between metabolic syndrome, fatty liver disease, and gastrointestinal tumors: a population-based study with external validation

**DOI:** 10.3389/fnut.2026.1706013

**Published:** 2026-03-04

**Authors:** Mengyao Zhang, Jie Song, Siqi Liu, Lanlan Yang, Qian Zhang, Zhenjing Jin

**Affiliations:** Department of Hepatopancreatobiliary Medicine, Digestive Disease Center, The Second Hospital of Jilin University, Changchun, Jilin, China

**Keywords:** fatty liver disease, gastrointestinal tumors, metabolic syndrome, population study, survival outcomes

## Abstract

**Background:**

Metabolic Syndrome (MetS), defined by central obesity and disturbances in glucose and lipid metabolism, has not been extensively validated in large national cohorts concerning its association with fatty liver disease (FLD), gastrointestinal tumors (GIT), and prognostic outcomes.

**Methods:**

A total of 24,434 adults from the 2003–2018 cycles of The National Health and Nutrition Examination Survey (NHANES) were included as the development cohort, with a validation cohort of 365 adults to verify key associations. MetS was diagnosed per NCEP-ATP III criteria across both cohorts. Weighted multivariate regression models assessed associations between MetS and FLD/GIT incidence, and Cox proportional hazards models evaluated survival risks. Three hierarchical models were constructed: Model 1 (unadjusted), Model 2 (adjusted for demographic confounders), and Model 3 (further adjusted for laboratory parameters).

**Results:**

In the development cohort, MetS patients exhibited a higher FLD prevalence (16.2% vs. 4.6%) and GIT incidence (1.25% vs. 0.57%). After full adjustment in Model 3, MetS remained a strong independent risk factor for FLD (OR = 3.889, 95% CI: 3.529–4.307) and GIT (OR = 2.456, 95% CI: 1.832–3.292). These associations were corroborated in the validation cohort, with adjusted ORs of 4.760 for FLD and 4.395 for GIT. Survival analysis indicated that MetS significantly reduced overall survival in the development cohort, with HRs for all-cause, cancer-specific, and cardiovascular mortality of 2.146, 1.941, and 2.572, respectively. Furthermore, the mortality risk was further elevated in FLD patients with MetS (all-cause mortality HR = 1.823). In the validation cohort, cardiovascular mortality risk was significant (HR = 3.902), while other survival outcomes did not reach statistical significance due to the small sample size. Sensitivity analysis using IDF criteria confirmed the robustness of these findings.

**Conclusion:**

This study confirms that MetS is strongly associated with FLD risk and GIT incidence, supporting early metabolic intervention to interrupt the progression of liver disease and tumors.

## Introduction

Metabolic syndrome (MetS) is a major public health challenge characterized by central obesity, hyperglycemia, hypertension, and dyslipidemia (1–3). While traditionally linked to cardiovascular disease, the systemic impact of MetS extends to other organ systems. Specifically, insulin resistance and chronic low-grade inflammation associated with MetS are increasingly implicated in the pathogenesis of non-cardiovascular chronic diseases.

Fatty liver disease (FLD) represents a primary hepatic manifestation of this metabolic dysfunction (4–6). Driven by insulin resistance and elevated free fatty acid flux, hepatocyte steatosis can progress to fibrosis, cirrhosis, and hepatocellular carcinoma (7). Although this biological link is biologically plausible, evidence from large-scale, representative national cohorts remains limited compared to clinical studies (8, 9). Consequently, the generalizability of these associations requires validation across diverse populations.

Gastrointestinal tumors (GIT) share a similar metabolic basis (10–14). Metabolic dysregulation may alter the intestinal microenvironment, promoting cell proliferation while inhibiting apoptosis (15). However, clinical data regarding MetS and GIT outcomes remain conflicting. While MetS is a recognized risk factor for tumor incidence, its influence on long-term survival in patients with established GIT is under-investigated and requires further clarification (16, 17).

Therefore, the present study aimed to investigate the associations of MetS with FLD and GIT using a large population-based development cohort and a validation cohort. We analyzed the prevalence of these conditions in individuals with MetS and further examined the potential impact of MetS on long-term survival outcomes. By integrating findings from both representative survey data and clinical records, this study seeks to clarify the epidemiological links between metabolic dysregulation and digestive health.

## Materials and methods

### Study population

**Figure 1:**
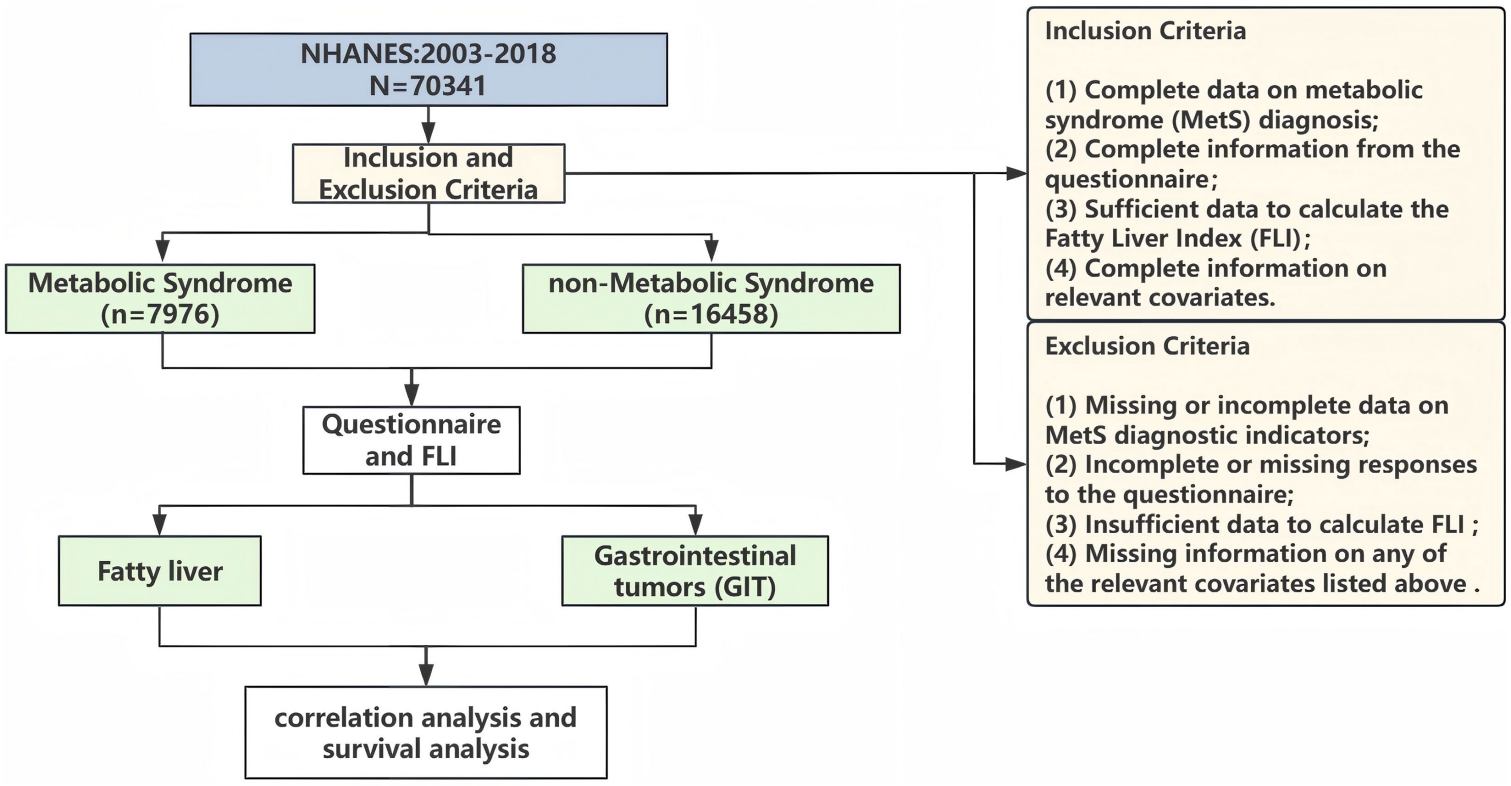
Flow chart of study population selection.

The National Health and Nutrition Examination Survey (NHANES) is a major program of the National Center for Health Statistics. It operates under the US Centers for Disease Control and Prevention. This population-based cross-sectional survey employs a stratified multistage sampling design and collects data in 2-year cycles. Data were collected through interviews, physical examinations, and laboratory tests, including demographic, dietary, examination, laboratory, and questionnaire data. The study was approved by the National Center for Health Statistics’ Research Ethics Review Committee, and all participants provided written informed consent. Detailed information is available at: https://www.cdc.gov/nchs/nhanes/index.html. A total of 70,341 participants from 2003 to 2018 were initially included. Exclusions were applied for missing MetS diagnosis data, incomplete cancer questionnaire results, missing relevant covariates, insufficient data for Fatty Liver Index (FLI) calculation, which resulted in a final sample of 24,434 participants (development cohort). To further verify the key associations identified in the development cohort, a validation cohort was incorporated, consisting of 365 adults (240 with non-MetS and 125 with MetS) from real-world clinical settings. The inclusion and exclusion process for the development cohort is detailed in Figure 1, and the validation cohort was selected based on consistent eligibility criteria to ensure comparability with the development cohort. The studies involving human participants were reviewed and approved by the Ethics Committee of The Second Hospital of Jilin University (Approval number: 2025–320). Given the retrospective nature of the study, the Ethics Committee waived the requirement for written informed consent from the participants.

### FLD assessment

For the development cohort, FLD was identified using the FLI, a well-validated non-invasive scoring model derived from routine clinical parameters. The FLI was calculated using the formula:

$\mathrm{FLI}=\frac{e^{\left[\begin{array}{l} 0.953\times \ln(\mathrm{TG})+0.139\times \mathrm{BMI}+0.718\times \\ \ln(\mathrm{GGT})+0.053\times \mathrm{WC}-15.745 \end{array}\right]}}{\left(1+e^{\left[\begin{array}{l} 0.953\times \ln(\mathrm{TG})+0.139\times \mathrm{BMI}+0.718\times \\ \ln(\mathrm{GGT})+0.053\times \mathrm{WC}-15.745 \end{array}\right]}\right)}\times 100.$

TG stands for triglycerides (mg/dL), BMI is body mass index (kg/m^2^), GGT refers to *γ*-glutamyl transferase (U/L), and WC is waist circumference (cm). An FLI of 30 or higher indicates FLD or high risk, while below 30 suggests no FLD. This threshold was chosen because mild hepatic steatosis often causes minimal metabolic changes, which can limit FLI sensitivity in detecting early mild FLD. By including those at “high risk of FLD” (FLI ≥ 30), this method broadens the scope for early metabolic intervention, addressing a gap in traditional criteria that typically only identify established FLD. FLI was selected as the primary screening tool for the development cohort due to its established accuracy and feasibility in large-scale epidemiological surveys where imaging data are not uniformly available.

In the validation cohort, FLD was diagnosed using abdominal ultrasound, the clinical standard for non-invasive assessment. Diagnosis criteria included increased hepatic parenchymal echo, blurred intrahepatic vascular structures, and reduced deep hepatic tissue echo. Two experienced radiologists conducted the evaluations, resolving any discrepancies with a third senior radiologist. This method reflects typical clinical practice, enabling verification of the development cohort’s findings using imaging rather than scoring models.

### MetS diagnosis

MetS was diagnosed according to the National Cholesterol Education Program Adult Treatment Panel III (NCEP-ATP III) criteria. Diagnosis required the presence of ≥3 criteria of the following: waist circumference ≥ 102 cm in men or ≥ 88 cm in women; fasting blood glucose ≥ 100 mg/dL or diagnosed diabetes; blood pressure ≥ 130/85 mmHg or diagnosed hypertension; triglycerides ≥ 150 mg/dL; high-density lipoprotein cholesterol < 40 mg/dL in men or < 50 mg/dL in women.

To ensure diagnostic consistency across cohorts and alignment with the reference database, the primary analysis applied the NCEP-ATP III criteria uniformly for MetS definition. In addition, a pre-specified sensitivity analysis was conducted to test the robustness of the findings under alternate diagnostic standards. For this sensitivity analysis, MetS was redefined according to the International Diabetes Federation (IDF) criteria, with Asian-specific waist circumference thresholds (≥ 90 cm for men and ≥ 80 cm for women) applied to the validation cohort of Chinese individuals.

### GIT ascertainment

GIT cases were identified using NHANES questionnaire data. Participants were asked, “What kind of cancer have you been diagnosed with?” Responses corresponding to GIT were coded and included. While GIT ascertainment was based on self-reported questionnaire data, this method has been previously validated in the NHANES population, showing high concordance with medical records and pathological reports. Given the sample size constraints for rare cancer subtypes (e.g., pancreatic and esophageal cancers) in both cohorts, GIT were analyzed as a composite group to ensure sufficient statistical power and the stability of multivariate models.

### Covariates

To minimize confounding bias, we selected covariates based on their established associations with both metabolic dysregulation and digestive pathology. Demographic and lifestyle variables were included to account for socioeconomic and behavioral differences, while laboratory parameters were selected to control for systemic inflammation and hepatic function. Consequently, the covariates encompassed age, sex, race, education level, poverty income ratio (PIR), smoking status, and alcohol consumption. Laboratory parameters included white blood cell count, platelet count, neutrophil count, lymphocyte count, creatinine, alanine aminotransferase (ALT), aspartate aminotransferase (AST), uric acid, and albumin levels. Data availability differed between the two cohorts. Comprehensive socioeconomic details, such as education, race, and PIR, are not routinely collected in clinical settings. Therefore, these specific variables were adjusted for exclusively in the development cohort. Specifically, education level was categorized as high school or below versus above high school, race was included, and PIR was originally classified into low-income (PIR ≤ 1.3), middle-income (PIR: 1.3–3.5), and high-income (PIR > 3.5) groups (18). Questionnaire data for the development cohort were gathered by NHANES researchers, while laboratory data for both cohorts were obtained through standardized procedures to ensure consistency and reliability.

### Statistical analysis

Continuous variables were summarized as weighted mean±SD for normal distributions and median (IQR) for non-normal distributions. Categorical variables were shown as frequencies (percentages). Associations between MetS and FLD/GIT incidence were assessed using weighted multivariate logistic regression. Survival outcomes were analyzed with Kaplan–Meier curves and Cox models. To address potential confounding arising from baseline differences between groups, we relied on multivariable adjustment in hierarchical models rather than propensity score matching. This approach allowed us to retain the full sample size and statistical power, particularly important for our validation cohort, while directly controlling for the same set of covariates that would have been used in a propensity score.

To evaluate the stability of the associations and identify the influence of different covariate clusters, we constructed three hierarchical regression models. Model 1 provided unadjusted estimates to establish the baseline association. Model 2 adjusted for demographic and lifestyle factors to control for background characteristics. Model 3 further adjusted for laboratory parameters to isolate the independent effect of MetS after accounting for biological confounders. Following the assessment for multicollinearity, the final set of laboratory covariates retained in the fully adjusted models (Model 3) included white blood cell count, ALT, albumin, creatinine, and platelet count. These parameters were selected for their established clinical relevance to the core pathophysiological pathways under investigation: systemic inflammation, hepatocellular injury and liver function, renal function, and broader metabolic and hematologic context. Furthermore, they are routinely available in both population-based and clinical settings, ensuring the practicality and generalizability of our models. After this selection, variance inflation factors (VIFs) for all retained covariates were confirmed to be below 2.0, indicating no substantial multicollinearity that would compromise the stability of the regression estimates. Results were reported as odds ratios (OR) or hazard ratios (HR) with 95% confidence intervals (CI). Additionally, a sensitivity analysis was performed by redefining MetS using the IDF criteria to evaluate the consistency of the observed associations. The proportional hazards assumption for all Cox regression models was rigorously validated using the Schoenfeld residual test. In instances where the large sample size of the development cohort led to significant *p*-values for specific covariates, the stability of the estimates was further confirmed through visual inspection of Schoenfeld residual plots to ensure that no meaningful violations of the proportionality assumption occurred.

### Model selection justification

Three hierarchical models were employed to sequentially control for potential confounders. Model 3 adjusted for routinely measured laboratory parameters to account for inflammation, organ function, and metabolic state. Standard Cox models were used for survival analysis. Time-dependent Cox models were not applicable as covariates were measured only at baseline. Competing risk models were considered but not employed in small subgroups due to limited event counts, which would compromise model stability.

## Results

### Baseline characteristics of study participants

A comparative analysis of the development cohort (*n* = 24,434) and validation (*n* = 365) cohorts revealed a consistent core phenotype associated with MetS, while highlighting expected and informative differences derived from their distinct population sources. In both cohorts, participants with MetS were older and exhibited a marked systemic inflammatory and metabolic perturbation, characterized by significantly elevated levels of white blood cells, neutrophils, liver enzymes (ALT, AST), and uric acid compared to non-MetS individuals (Table 1).

**Table 1:** Baseline characteristics of development cohort.

Characteristic	MetS		*p* value
	Yes (*n* = 7,976)	No (*n* = 16,458)	
Age, years (Mean ± SD)	56.13 ± 16.00	46.49 ± 17.80	<0.001
Sex, (male/female, n)	5052/2924	7064/9394	<0.001
Race (Hispanic/Non-Hispanic, n)	2125/5851	3652/12806	<0.001
Educational level, n (%)			
Below high school	2,321 (29.10)	3,455 (20.99)	<0.001
High school or equivalent	2003 (25.11)	3,686 (23.30)	
Above high school	3,652 (45.79)	9,317 (56.61)	
Ratio of family income to poverty, n (%)			
≤1.3	2,632 (33.00)	4,956 (30.11)	<0.001
1.3–3.5	3,117 (39.08)	6,070 (36.88)	
>3.5	2,227 (27.92)	5,432 (33.01)	
Smoking (yes/no, n)	4198/3778	7052/9460	<0.001
Alcohol use (yes/no, n)	5983/1993	12,492/3966	0.13
White Blood Cell, 1,000 cells/μL	7.40 (6.10–8.80)	6.70 (5.60–8.10)	<0.001
Neutrophil Count, 1,000 cells/μL	4.30 (3.40–5.40)	3.90 (3.00–5.00)	<0.001
Platelet Count, 1,000 cells/μL	237 (200.0–281.00)	240.00 (205.00–284.00)	<0.001
Lymphocyte Count, 1,000 cells/μL	2.10 (1.70–2.70)	2.00 (1.60–2.50)	<0.001
Alanine Aminotransferase, U/L	23.0 (18.0–32.0)	19.0 (15.0–26.0)	<0.001
Aspartate Aminotransferase, U/L	24.0 (20.0–29.0)	22.0 (19.0–26.0)	<0.001
Creatinine, μmol/L	80.44(68.07–96.36)	72.49(61.88–87.52)	<0.001
Albumin, g/L	4.20(4.00–4.40)	4.20(4.00–4.50)	<0.001
Uric Acid, μmol/L	350.90 (297.40–410.40)	303.30 (249.80–362.80)	<0.001
GIT (yes/no)	100/7876	95/16363	<0.001
FLD (yes/no)	1288/6688	753/15705	<0.001

MetS, metabolic syndrome; FLD, fatty liver disease; GIT, gastrointestinal tumors; SD, standard deviation.

Notably, the absolute prevalence of the primary outcomes was higher in the validation cohort (MetS subgroup: FLD 22.4%, GIT 12.0%) than in the population-based development cohort (MetS subgroup: FLD 16.2%, GIT 1.25%). This gradient in disease burden is anticipated and reinforces the clinical relevance of our findings; the validation cohort, enriched with patients seeking medical care, naturally represents a segment of the MetS population with more advanced pathological manifestations, whereas the development cohort reflects the broader disease spectrum in the general population. Furthermore, differences in available sociodemographic variables reflect the distinct settings (US national survey vs. Chinese clinical center) and do not pertain to the core metabolic dysregulation under investigation. Minor inconsistencies in specific laboratory parameters, such as platelet count, are likely attributable to cohort-specific factors including sample size and measurement context.

Critically, the concordance in the fundamental metabolic and inflammatory profile of MetS across both cohorts underscores the robustness of this phenotype. The findings from both cohorts converged, demonstrating a strong association between MetS and increased odds of FLD and GIT. This consistency remained evident despite differences in cohort origins, disease prevalence, and social determinants. Such robustness strengthens the generalizability of our conclusions. It suggests that the observed relationships are driven by the underlying biology of MetS rather than cohort-specific artifacts (Table 2).

**Table 2:** Baseline characteristics of validation cohort.

Characteristic	MetS		*p*-value
	Yes (*n* = 125)	No (*n* = 240)	
Age, years (Mean ± SD)	54.03 ± 15.81	43.94 ± 16.71	<0.001
Sex, (male/female, n)	85/40	102/138	<0.001
Smoking (yes/no, n)	56/69	91/149	0.25
Alcohol use (yes/no, n)	93/32	179/61	1.00
White Blood Cell, 1,000 cells/μL	7.40(6.40–9.00)	6.50 (5.50–7.73)	<0.001
Neutrophil Count, 1,000 cells/μL	4.40 (3.60–5.50)	3.75 (2.98–4.70)	<0.001
Platelet Count, 1,000 cells/μL	237 (200.0–281.00)	240.00 (205.00–284.00)	0.76
Lymphocyte Count, 1,000 cells/μL	2.10 (1.70–2.60)	2.00 (1.60–2.40)	0.03
Alanine Aminotransferase, U/L	23.00 (19.00–29.00)	19.00 (15.00–25.00)	0.09
Aspartate Aminotransferase, U/L	23.00 (20.00–27.00)	22.00 (19.00–27.00)	0.45
Creatinine, μmol/L	80.44(68.07–96.36)	72.49 (61.88–85.75)	<0.001
Albumin, g/L	4.20(4.00–4.40)	4.20(4.00–4.50)	0.73
Uric Acid, μmol/L	356.90(297.40–410.40)	300.35 (237.9–368.8)	<0.001
GIT (yes/no)	15/110	9/231	0.01
FLD (yes/no)	28/97	17/223	<0.001

MetS, metabolic syndrome; FLD, fatty liver disease; GIT, gastrointestinal tumors; SD, standard deviation.

### Association of MetS with the incidence of FLD and GIT

Prior to conducting the survival analysis, diagnostic testing using Schoenfeld residuals confirmed that the primary association between MetS and mortality adhered to the proportional hazards assumption (*p* = 0.131 for MetS in the fully adjusted model). While the global test was significant due to the high statistical power inherent in the large sample size (*n* = 24,434), the overall model performance and HR estimations were found to be stable and valid for clinical interpretation. The association of MetS with FLD and GIT was evaluated using weighted multivariate logistic regression across three hierarchical models: unadjusted, adjusted for demographics and lifestyle factors, and fully adjusted for demographics, lifestyle factors, and laboratory parameters. The analysis confirmed that MetS significantly increased the incidence of FLD and GIT in both cohorts (refer to Table 3). In the development cohort, participants with MetS exhibited a 3.5-fold higher incidence of FLD compared to those without MetS (16.2% vs. 4.6%, *p* < 0.05). Even after full adjustment in Model 3, MetS persisted as a strong independent risk factor (OR = 3.889, 95% CI: 3.529–4.307, *p* < 0.05). This association was corroborated by the validation cohort. In this group, the incidence of FLD was 3.1-fold higher in participants with MetS (22.4% vs. 7.1%, *p* < 0.05). The fully adjusted model yielded an OR of 4.760 (95% CI: 2.332–9.713, *p* < 0.05). These results underscore the consistent role of MetS as a risk factor for FLD.

**Table 3:** Association of MetS with the onset of FLD and GIT.

FLD	MetS	Total, n	Events, n (%)	Model 1 OR (95% CI)	Model 2 Adjusted OR (95% CI)	Model 3 Adjusted OR (95% CI)
Development cohort	No	16,458	753, 4.60%	1 (Reference)	1 (Reference)	1 (Reference)
	Yes	7,976	1,288, 16.20%	4.017 (3.656–4.414)	5.044 (4.546–5.595)	3.889 (3.529–4.307)
Validation cohort	No	240	17, 7.10%	1 (Reference)	1 (Reference)	1 (Reference)
	Yes	125	28, 22.40%	3.787 (1.981–7.239)	4.710 (2.335–9.499)	4.760 (2.332–9.713)
GIT						
Development cohort	No	16,458	95, 0.57%	1 (Reference)	1 (Reference)	1 (Reference)
	Yes	7,976	100, 1.25%	2.187 (1.649–2.900)	1.389 (1.039–1.858)	2.456 (1.832–3.292)
Validation cohort	No	240	9, 3.75%	1 (Reference)	1 (Reference)	1 (Reference)
	Yes	125	15, 12.00%	3.500 (1.485–8.246)	3.291 (1.349–8.030)	4.395 (1.752–11.024)

MetS, metabolic syndrome; FLD, fatty liver disease; GIT, gastrointestinal tumors; OR, odds ratio; CI, confidence interval. Model 1: Unadjusted. Model 2: Adjusted for age, sex, race, education level, PIR, smoking, and alcohol use. Model 3: Adjusted for Model 2 plus white blood cell count, ALT, albumin, creatinine, and platelet count.

In the development cohort, participants with MetS exhibited a 2.2-fold higher incidence of GIT (1.25% vs. 0.57%, *p* < 0.05). This significance was preserved upon full adjustment (Model 3), yielding an OR of 2.456 with a 95% CI of 1.832–3.292 (*p* < 0.05). The validation cohort demonstrated an even more pronounced effect. MetS participants showed a 3.2-fold increase in GIT incidence (12.0% vs. 3.75%, *p* < 0.05). Furthermore, the fully adjusted analysis yielded an OR of 4.395 (95% CI: 1.752–11.024, *p* < 0.05). This heightened effect observed in the validation cohort may be attributable to its real-world composition, which likely encompasses a greater number of individuals with pre-existing metabolic or gastrointestinal vulnerabilities.

### MetS worsens survival rate but has no significant impact on the prognosis of GIT

**Figure 2:**
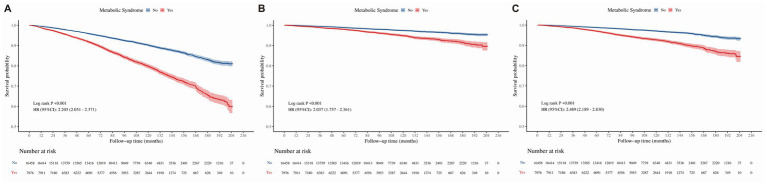
Kaplan–Meier survival curves for the association between MetS and mortality in the overall study population. **(A)** All-cause mortality. **(B)** Cancer-specific mortality. **(C)** Cardiovascular mortality. Log-rank tests were used to compare survival differences between groups. Hazard ratios (HRs) and 95% confidence intervals (CIs) were derived from unadjusted Cox proportional hazards models. The number of subjects at risk during follow-up is shown below each graph. MetS, metabolic syndrome; FLD, fatty liver disease; GIT, gastrointestinal tumors; HR, hazard ratio; CI, confidence interval.

Cox proportional hazards models, stratified by FLD and GIT status, were employed to assess the impact of MetS on survival outcomes, including all-cause mortality, cancer-specific mortality, and cardiovascular mortality (refer to Tables 4–7). Kaplan–Meier survival analysis demonstrated distinct separation in survival curves between the MetS and non-MetS groups (Figure 2). Consistent with this, the unadjusted Cox regression (Model 1) showed that MetS increased the risk of all-cause mortality by 2.2-fold (HR = 2.205, 95% CI: 2.051–2.371), cancer-specific mortality by 2.0-fold (HR = 2.037, 95% CI: 1.757–2.361), and cardiovascular mortality by 2.5-fold (HR = 2.489, 95% CI: 2.189–2.830). After adjusting for potential confounders, the development cohort revealed that MetS was significantly associated with increased risks of all-cause mortality (fully adjusted HR = 2.146, 95% CI: 1.993–2.312, *p* < 0.05), cancer-specific mortality (fully adjusted HR = 1.941, 95% CI: 1.671–2.255, *p* < 0.05), and cardiovascular mortality (fully adjusted HR = 2.572, 95% CI: 1.253–2.936, *p* < 0.05). Conversely, the validation cohort exhibited limited statistical power due to a small sample size, with only cardiovascular mortality reaching statistical significance (fully adjusted HR = 3.902, 95% CI: 1.123–13.559, *p* < 0.05). In the validation cohort, although all-cause mortality (fully adjusted HR = 1.770, 95% CI: 0.968–3.261) and cancer-specific mortality (fully adjusted HR = 1.887, 95% CI: 0.508–6.952) did not reach statistical significance (*p* > 0.05), the direction and magnitude of these risk estimates were highly consistent with those observed in the larger development cohort, suggesting a similar biological trend that may be confirmed with a larger sample size.

**Table 4:** Association of MetS on survival in the overall population.

All-cause mortality						
Overall population	MetS	Total, n	Events, n (%)	Model 1 OR (95% CI)	Model 2 Adjusted HR (95% CI)	Model 3 Adjusted HR (95% CI)
Development cohort	No	16,458	1,433, 8.70%	1 (Reference)	1 (Reference)	1 (Reference)
	Yes	7,976	1,513, 18.97%	2.205 (2.051–2.371)	1.244 (1.156–1.339)	2.146 (1.993–2.312)
Validation cohort	No	240	22, 9.17%	1 (Reference)	1 (Reference)	1 (Reference)
	Yes	125	24, 19.20%	2.202 (1.226–3.902)	1.439 (0.857–3.707)*	1.770 (0.968-3.261)*
Cancer-specific mortality						
Development cohort	No	16,458	377, 2.29%	1 (Reference)	1 (Reference)	1 (Reference)
	Yes	7,976	333, 4.18%	2.037 (1.757–2.361)	1.164 (1.001–1.354)	1.941 (1.671–2.255)
Validation cohort	No	240	5, 2.08%	1 (Reference)	1 (Reference)	1 (Reference)
	Yes	125	5, 4.00%	2.020 (0.585–6.977)*	1.324 (0.377-4.651)*	1.887 (0.508-6.952)*
Cardiovascular mortality						
Development cohort	No	16,458	453, 2.75%	1 (Reference)	1 (Reference)	1 (Reference)
	Yes	7,976	482, 6.04%	2.489 (2.189–2.830)	1.380 (1.211–1.573)	2.572 (1.253–2.936)
Validation cohort	No	240	4, 1.67%	1 (Reference)	1 (Reference)	1 (Reference)
	Yes	125	9, 7.20%	4.488 (1.382–14.575)	3.132 (0.943–10.400)*	3.902 (1.123-13.559)

*p > 0.05 and thus this value is not statistically significant. MetS, metabolic syndrome; FLD, fatty liver disease; GIT, gastrointestinal tumors; HR, hazard ratio; CI, confidence interval. Model 1: Unadjusted. Model 2: Adjusted for age, sex, race, education level, PIR, smoking, and alcohol use. Model 3: Adjusted for Model 2 plus white blood cell count, ALT, albumin, creatinine, and platelet count.

**Table 5:** Association of MetS on survival in population with FLD.

FLD					
MetS	Total, n	All-cause mortality, n (%)	Model 1 OR (95% CI)	Model 2 Adjusted HR (95% CI)	Model 3 Adjusted HR (95% CI)
No	753	55, 7.30%	1 (Reference)	1(Reference)	1 (Reference)
Yes	1,288	183, 14.21%	2.146 (1.587–2.902)	1.582 (1.144–2.187)	1.823 (1.323–2.511)
		Cancer-specific mortality, n (%)			
No	753	9, 1.20%	1 (Reference)	1 (Reference)	1 (Reference)
Yes	1,288	34, 2.64%	2.398 (1.150–5.001)	1.312 (0.593–2.902)*	2.480 (1.148–5.358)
		Cardiovascular mortality, n (%)			
No	753	21, 2.79%	1 (Reference)	1 (Reference)	1 (Reference)
Yes	1,288	65, 5.05%	1.997 (1.220–3.269)	1.560 (0.920–2.648)*	2.143 (1.278–3.953)

*p > 0.05 and thus this value is not statistically significant. MetS, metabolic syndrome; FLD, fatty liver disease; GIT, gastrointestinal tumors; HR, hazard ratio; CI, confidence interval. Model 1: Unadjusted. Model 2: Adjusted for age, sex, race, education level, PIR, smoking, and alcohol use. Model 3: Adjusted for Model 2 plus white blood cell count, ALT, albumin, creatinine, and platelet count.

**Table 6:** Association of MetS on survival in population with non-FLD.

All-cause mortality						
Non-FLD	MetS	Total, n	Events, n (%)	Model 1 OR (95% CI)	Model 2 Adjusted HR (95% CI)	Model 3 Adjusted HR (95% CI)
Development cohort	No	15,705	1,458, 9.28%	1 (Reference)	1 (Reference)	1 (Reference)
	Yes	6,688	1,250, 18.69%	2.259 (2.094–2.437)	1.203 (1.114–1.300)	2.211 (2.047–2.389)
Validation cohort	No	223	22, 9.87%	1 (Reference)	1 (Reference)	1 (Reference)
	Yes	97	23, 23.71%	2.641 (1.472–4.741)	1.665 (0.916–1.985)*	2.182 (1.176–4.048)
Cancer-specific mortality						
Development cohort	No	15,705	368, 2.34%	1 (Reference)	1 (Reference)	1 (Reference)
	Yes	6,688	299, 4.47%	2.120 (1.820–2.470)	1.168 (0.999–1.366)*	2.042 (1.750–2.383)
Validation cohort	No	223	5, 2.24%	1 (Reference)	1 (Reference)	1 (Reference)
	Yes	97	5, 5.15%	2.498 (0.723–8.630)*	1.561 (0.441-5.526)*	2.490 (0.184-8.733)*
Cardiovascular mortality						
Development cohort	No	15,705	432, 2.75%	1 (Reference)	1 (Reference)	1 (Reference)
	Yes	6,688	299, 4.47%	2.556 (2.234–2.925)	1.325 (1.156–1.519)	2.617 (2.279–3.005)
Validation cohort	No	223	4, 1.79%	1 (Reference)	1 (Reference)	1 (Reference)
	Yes	97	8, 8.25%	5.088 (1.530–16.921)	3.079 (0.902–10.513)*	4.269 (1.175-15.504)

*p > 0.05 and thus this value is not statistically significant. MetS, metabolic syndrome; FLD, fatty liver disease; GIT, gastrointestinal tumors; HR, hazard ratio; CI, confidence interval. Model 1: Unadjusted. Model 2: Adjusted for age, sex, race, education level, PIR, smoking, and alcohol use. Model 3: Adjusted for Model 2 plus white blood cell count, ALT, albumin, creatinine, and platelet count.

**Table 7:** Association of MetS on survival in population with GIT.

GIT					
MetS	Total, n	All-cause mortality, n (%)	Unadjusted HR	Model 2 Adjusted HR (95% CI)	Model 3 Adjusted HR (95% CI)
No	95	37, 38.94%	1 (Reference)	1 (Reference)	1 (Reference)
Yes	100	48, 48.00%	1.280 (0.840–1.950)*	-	-
		Cancer-specific mortality, n (%)			
No	95	8, 8.42%	1 (Reference)	1 (Reference)	1 (Reference)
Yes	100	15, 15.00%	1.863 (0.789–4.397)*	-	-
		Cardiovascular mortality, n (%)			
No	95	9, 9.47%	1 (Reference)	1 (Reference)	1 (Reference)
Yes	100	16, 16.00%	1.836 (0.810–4.161)*	-	-

*p > 0.05 and thus this value is not statistically significant. MetS, metabolic syndrome; FLD, fatty liver disease; GIT, gastrointestinal tumors; HR, hazard ratio; CI, confidence interval. Model 1: Unadjusted. Model 2: Adjusted for age, sex, race, education level, PIR, smoking, and alcohol use. Model 3: Adjusted for Model 2 plus white blood cell count, ALT, albumin, creatinine, and platelet count.

**Figure 3:**
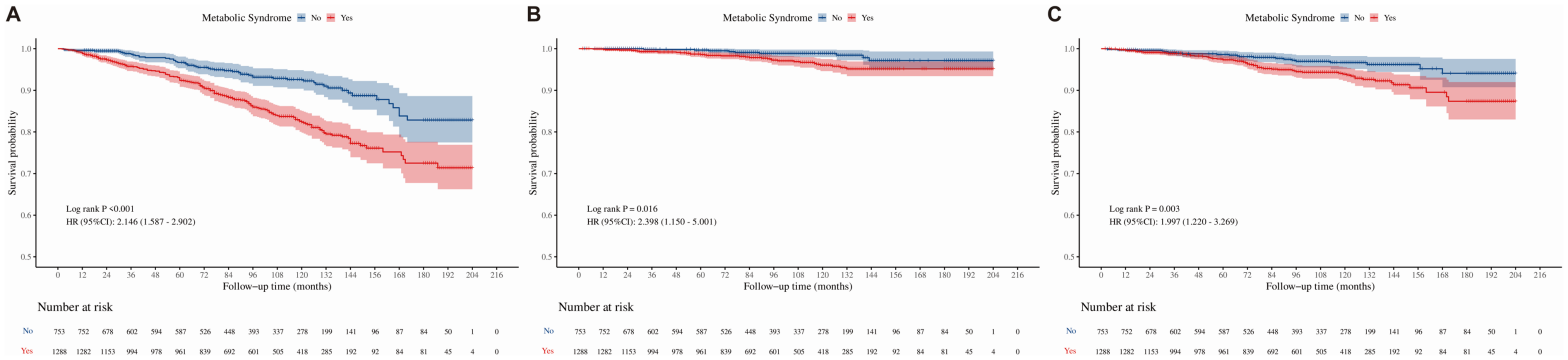
Kaplan–Meier survival curves for the association between MetS and mortality in the subgroup of participants with FLD. **(A)** All-cause mortality. **(B)** Cancer-specific mortality. **(C)** Cardiovascular mortality. Hazard ratios (HRs) and 95% confidence intervals (CIs) were derived from unadjusted Cox proportional hazards models. MetS, metabolic syndrome; FLD, fatty liver disease; GIT, gastrointestinal tumors; HR, hazard ratio; CI, confidence interval.

**Figure 4:**
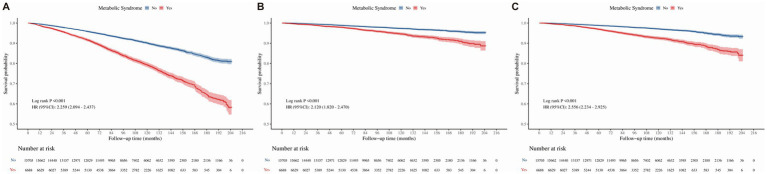
Kaplan–Meier survival curves for the association between MetS and mortality in the subgroup of participants without FLD. **(A)** All-cause mortality. **(B)** Cancer-specific mortality. **(C)** Cardiovascular mortality. Hazard ratios (HRs) and 95% confidence intervals (CIs) were derived from unadjusted Cox proportional hazards models. MetS, metabolic syndrome; FLD, fatty liver disease; GIT, gastrointestinal tumors; HR, hazard ratio; CI, confidence interval.

**Figure 5:**
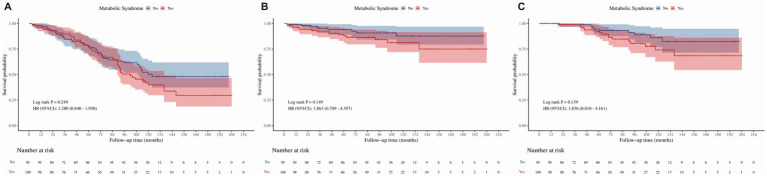
Kaplan–Meier survival curves for the association between MetS and mortality in the subgroup of participants with GIT. **(A)** All-cause mortality. **(B)** Cancer-specific mortality. **(C)** Cardiovascular mortality. Hazard ratios (HRs) and 95% confidence intervals (CIs) were derived from unadjusted Cox proportional hazards models. MetS, metabolic syndrome; FLD, fatty liver disease; GIT, gastrointestinal tumors; HR, hazard ratio; CI, confidence interval.

Stratification based on FLD status revealed significant effect modifications. As shown in Figure 3, MetS significantly worsened survival outcomes in patients with FLD. In the unadjusted analysis, MetS was associated with elevated risks for all-cause (HR = 2.146), cancer-specific (HR = 2.398), and cardiovascular mortality (HR = 1.997). Upon full adjustment, participants with MetS and FLD exhibited higher rates of all-cause mortality (fully adjusted HR = 1.823, 95%CI: 1.323–2.511, *p* < 0.05) and cardiovascular mortality (fully adjusted HR = 2.143, 95% CI: 1.278–3.953, *p* < 0.05), with a statistically significant increase in cancer-specific mortality (fully adjusted HR = 2.480, 95% CI: 1.148–5.358, *p* < 0.05). Conversely, among participants without FLD, the survival curves similarly indicated poor prognosis for the MetS group (Figure 4). The unadjusted model confirmed significant associations with all-cause (HR = 2.259), cancer-specific (HR = 2.120), and cardiovascular mortality (HR = 2.556). Even after adjustment, MetS remained a significant predictor of mortality across both cohorts. The development cohort demonstrated significant elevations in all three mortality endpoints (fully adjusted HRs: 2.211 for all-cause, 2.042 for cancer-specific, and 2.617 for cardiovascular mortality; all *p* < 0.05). The validation cohort corroborated these findings, showing significant increases in all-cause mortality (fully adjusted HR = 2.182, 95% CI: 1.176–4.048, *p* < 0.05) and cardiovascular mortality (fully adjusted HR = 4.269, 95% CI: 1.175–15.504, *p* < 0.05). In participants already diagnosed with GIT, Kaplan–Meier curves suggested a trend toward lower survival in the MetS group (Figure 5). However, in the unadjusted Cox analysis, these associations did not reach statistical significance for all-cause (HR = 1.280, *p* > 0.05) or cancer-specific mortality (HR = 1.863, *p* > 0.05). Similarly, in the adjusted models, MetS showed a trend toward increased mortality (e.g., HR = 1.863 for cancer-specific mortality), though these findings did not reach statistical significance. This lack of significance is likely attributable to the limited number of events (*n* = 48 deaths in the MetS group) in this specific subpopulation, which restricts the statistical power to detect smaller but potentially clinical meaningful differences.

Due to the limited sample size and the absence of time-varying covariate data, more complex models such as time-dependent Cox or competing risk models were not employed.

### Sensitivity analysis

To assess the robustness of our primary findings, a pre-specified sensitivity analysis was conducted by redefining MetS according to the IDF criteria (with Asian-specific waist circumference thresholds for the validation cohort). The results consistently confirmed the core associations observed in the primary analysis (Supplementary Tables S1, S2).

Regarding the incidence of FLD and GIT, in the development cohort, IDF-defined MetS was significantly associated with an increased risk of FLD (adjusted OR = 4.833, 95% CI: 4.307–5.424) and GIT (adjusted OR = 2.413, 95% CI: 1.780–3.217), with slightly stronger effect sizes for FLD compared to the primary NCEP-ATP III definition (adjusted OR = 3.889 for FLD; 2.456 for GIT). In the validation cohort, the associations remained significant for both FLD (adjusted OR = 4.320, 95% CI: 2.074–8.997) and GIT (adjusted OR = 4.257, 95% CI: 1.648–10.991), which were comparable to the primary results (adjusted OR = 4.760 for FLD; 4.395 for GIT).

For survival outcomes, in the development cohort, IDF-defined MetS was associated with elevated all-cause mortality (adjusted HR = 1.730, 95% CI: 1.602–1.868), cancer-specific mortality (adjusted HR = 1.591, 95% CI: 1.363–1.858), and cardiovascular mortality (adjusted HR = 2.097, 95% CI: 1.827–2.406). These effect sizes were moderately lower than those in the primary analysis (adjusted HR = 2.146 for all-cause mortality; 1.941 for cancer-specific mortality; 2.572 for cardiovascular mortality) but retained statistical significance. In the validation cohort, IDF-defined MetS was significantly associated with all-cause mortality (adjusted HR = 3.071, 95% CI: 1.583–5.957) and showed a non-significant trend toward increased cancer-specific (adjusted HR = 8.284, 95% CI: 1.606–42.735, *p* > 0.05) and cardiovascular mortality (adjusted HR = 4.006, 95% CI: 0.974–14.472, *p* > 0.05), consistent with the primary findings of limited statistical power due to the small sample size.

Notably, despite minor variations in effect sizes for certain outcomes between the NCEP-ATP III and IDF criteria, the direction of association and statistical significance of all core findings remained consistent—including the positive links between MetS and FLD/GIT incidence, as well as the elevated risks of all-cause, cancer-specific, and cardiovascular mortality associated with MetS. This consistency indicates that our key conclusions are robust to variations in MetS diagnostic standards, and the observed differences in effect magnitudes reflect nuanced differences in how each criterion operationalizes metabolic dysregulation rather than a fundamental shift in the underlying biological relationship.

## Discussion

The results of this study demonstrated that MetS was significantly associated with an increased incidence of FLD (adjusted ORs: 3.889–4.760) and GIT (adjusted ORs: 2.456–4.395). However, contrary to our hypothesis, no significant impact of MetS on the prognosis of patients with GIT was observed (*p* > 0.05).

### Verification that MetS is a risk factor for FLD

Drawing on development cohort, this study substantiates that MetS increases the risk of FLD by nearly 4-fold (adjusted OR = 3.889, 95% CI: 3.529–4.307), aligning with previous research findings. The fundamental pathophysiological mechanisms of MetS—namely, insulin resistance, abdominal obesity, and dyslipidemia—contribute to the onset of FLD through multiple pathways (19, 20). Insulin resistance promotes the catabolism of adipose tissue, leading to an increased influx of free fatty acids and their subsequent accumulation in the liver, thus establishing a lipotoxic environment. Moreover, hyperglycemia and hyperinsulinemia activate hepatic inflammatory pathways, inducing oxidative stress and hepatocyte damage, which expedite the progression of steatosis. Furthermore, dysbiosis in the intestinal microbiota, frequently observed in patients with MetS, may augment the hepatic metabolic burden via the “gut-liver axis,” thereby exacerbating the progression of FLD (21–23).

The findings suggest that early intervention in MetS, including weight reduction, improvement of insulin sensitivity, and regulation of blood lipid levels, may represent a critical strategy for the prevention of FLD. Previous research highlights that lifestyle-induced weight loss significantly reduces hepatic fat accumulation, which has important implications for the primary prevention of FLD in individuals with MetS. It is essential for clinicians to carefully monitor the risk of FLD in patients with metabolic disorders to prevent disease progression (5, 24).

It is noteworthy that although FLI performance may vary in individuals with extreme metabolic profiles, our study addressed this by incorporating a validation cohort diagnosed via abdominal ultrasound. The high degree of concordance between the FLI-based findings and ultrasound-confirmed results reinforces the validity of the observed association between MetS and FLD risk.

### Association between MetS and the incidence of GIT

This study links MetS to a higher risk of GIT, supporting the “metabolic disorder–chronic inflammation–tumorigenesis” theory. MetS is associated with GIT development through specific mechanisms. Chronic inflammation and oxidative stress are significant contributors to the pathogenesis of various malignancies. Abdominal obesity and hyperglycemia prompt adipocytes to secrete pro-inflammatory cytokines, thereby establishing a chronic low-grade inflammatory microenvironment that can lead to DNA damage and promote aberrant cell proliferation. Insulin, as a critical growth factor, is instrumental in tumorigenesis, particularly in cancers linked to metabolic dysfunction. In the context of insulin resistance and metabolic dysfunction-associated steatohepatitis (MASH), the PI3K/Akt/mTOR signaling pathway undergoes pathological “imprinting,” which transforms it into a persistent oncogenic signaling hub. This altered pathway is responsive to metabolic disturbances such as hyperinsulinemia, consequently driving tumor cell proliferation, facilitating metabolic reprogramming, and contributing to the development of an immunosuppressive tumor microenvironment. This mechanism is particularly evident in hepatocellular carcinoma associated with insulin resistance (25).

Dysregulation of intestinal flora induced by MetS critically disrupts intestinal homeostasis. This shift is characterized by diminished production of protective short-chain fatty acids (SCFAs) alongside an increased release of bacterial endotoxins, collectively promoting a pro-inflammatory and pro-carcinogenic microenvironment (26, 27).

While we observed a significant overall association, the mechanisms likely vary across GIT subtypes. For instance, in colorectal cancer, MetS-related hyperinsulinemia may directly promote tumor cell proliferation via the IGF-1 axis (28). In contrast, the link to hepatocellular carcinoma is more likely mediated through the progression of FLD to chronic inflammation and cirrhosis (29). Although our combined analysis focused on shared metabolic pathways like chronic inflammation, we acknowledge that specific oncogenic drivers may be masked by this aggregation.

### Possible explanations for the lack of significant association between MetS and GIT

In our survival analysis, the adjusted HR for cancer-specific mortality in GIT patients with MetS was 1.863 (95% CI: 0.789–4.397, *p* > 0.05). The absence of a significant association between MetS and prognosis in patients with established GIT, while seemingly inconsistent with its role in tumor incidence, may be understood through the interplay of dominant oncological drivers and integrated clinical care. Once a malignancy is diagnosed, tumor biology—including its molecular profile, histological grade, and stage—assumes a primary role in determining outcome, an influence that can eclipse that of systemic metabolic factors. Moreover, MetS itself is a heterogeneous cluster of abnormalities; individual components may have divergent effects on cancer survival. For instance, hypertension or diabetes could increase susceptibility to treatment-related complications, whereas obesity might, in some contexts, be associated with greater metabolic reserves and better treatment tolerance. When aggregated into a single syndrome, these opposing influences may counterbalance each other, resulting in a net neutral association. We postulate that in contemporary oncology practice, the routine management of metabolic comorbidities may attenuate any independent prognostic impact of MetS. Thus, the observed neutral prognostic association likely reflects the overriding importance of tumor-intrinsic biology.

### Sensitivity analysis

To ensure the robustness of our primary findings and address potential concerns regarding the choice of diagnostic criteria, we conducted a sensitivity analysis redefining MetS using the IDF criteria. The results of this analysis provide strong validity for our conclusions. Specifically, using IDF criteria, MetS remained significantly associated with FLD (adjusted OR = 4.833) and GIT (adjusted OR = 2.413). Crucially, the direction and statistical significance of all key associations—between MetS and the incidence of FLD and GIT, as well as with all-cause and cause-specific mortality—were entirely consistent between the NCEP-ATP III and IDF definitions across both cohorts.

The variation in the estimated effects is anticipated because the NCEP-ATP III and IDF criteria apply different thresholds, particularly for waist circumference, and therefore capture overlapping but not identical populations. The IDF criteria, with its emphasis on population-specific waist circumference, might identify a slightly different, and potentially more metabolically severe, subset within the broader MetS phenotype. That the core associations not only persist but remain strong under this alternative definition underscores that the relationship is driven by the underlying metabolic dysfunction itself, rather than being an artifact of a specific operationalization.

Importantly, this consistency across diagnostic frameworks significantly strengthens the validity and generalizability of our study. It demonstrates that the identified risks of FLD and GIT, as well as the adverse survival outcomes associated with MetS, are robust findings not contingent upon a single, arbitrary diagnostic threshold. This reinforces the public health and clinical implication that interventions targeting core metabolic disturbances, rather than merely meeting a specific diagnostic checklist, are warranted for risk mitigation.

### Clinical implications

Management of MetS must extend beyond cardiovascular health to include digestive protection. We recommend routine screening for FLD using non-invasive tools, such as FLI or abdominal ultrasound. Clinicians should note that the presence of FLD in MetS patients indicates a significantly higher risk for GIT. Therefore, closer surveillance for tumors is warranted in this high-risk subgroup. Therapeutically, interventions must target core metabolic disturbances rather than isolated symptoms. Improving insulin sensitivity and reducing inflammation are critical steps to protect liver function and reduce the incidence of GIT.

### Limitations and future perspectives

While this study offers significant clinical insights, several limitations should be acknowledged. First and foremost, the cross-sectional design of the NHANES study limits our ability to infer causal relationships or determine the exact chronological order between MetS and the development of FLD or GIT. While our findings show strong statistical associations, prospective longitudinal studies are required to confirm the causative role of metabolic dysregulation in these diseases.

Second, GIT were analyzed as a composite group without stratification by anatomic site or histopathology. This approach may obscure potential differences in how MetS influences distinct tumor types. For instance, the well-established association of colorectal cancer with insulin resistance and gut microbiota could be diluted in a combined analysis, while the link between hepatocellular carcinoma and FLD might be underrepresented. Therefore, future investigations should aim for larger, multi-center cohorts that allow for site-specific analyses to develop tailored metabolic interventions.

Third, the control for confounding was incomplete owing to constraints in the available datasets; key clinicopathological variables such as tumor grade, stage, and treatment regimens could not be included, which may affect the precision of the estimated associations. Furthermore, the study was limited to identifying statistical associations and did not investigate the underlying molecular mechanisms. To bridge this gap, future studies should integrate molecular biology techniques to explore the potential mechanisms—such as the “gut-liver axis” or inflammatory pathways—connecting metabolic disorders to tumor occurrence.

Lastly, we relied on comprehensive multivariable adjustment rather than propensity score matching to preserve statistical power. Despite these limitations, this study provides a foundational understanding that warrants further verification through rigorously designed prospective trials.

## Conclusion

This study confirms that MetS is an important risk factor for the occurrence of FLD and GIT, but its independent prognostic impact appears limited in patients with established GIT. Future studies can adopt multi-center prospective designs, expand the sample size and extend the follow-up time, and combine molecular biology techniques to explore the potential mechanism of metabolic disorders affecting tumor occurrence. In clinical practice, attention should be paid to the early screening and intervention of patients with MetS to reduce the risk of GIT.

## Data Availability

The datasets presented in this article are not readily available because due to the requirements of the Ethics Committee, the original data of this article cannot be made public. Requests to access the datasets should be directed to jinzj@jlu.edu.cn.
