# Supplementary material for: Association between metabolic syndrome, fatty liver disease, and gastrointestinal tumors: a population-based study with external validation
Source: Front Nutr. 2026 Mar 4;13:1706013. doi: 10.3389/fnut.2026.1706013 (PMC12997174; doi:10.3389/fnut.2026.1706013)
Supplement: Supplementary file 1 [file Table_1.doc]

| **Fatty liver** | **Mets** | **Total, n** | **Events, n (%)** | **Model** 1 OR (95% CI) | **Model** 2 Adjusted OR (95% CI) | **Model** 3 Adjusted **OR (95% CI)** |
| --- | --- | --- | --- | --- | --- | --- |
| **Development cohort** | **No** | 14948 | 434, 2.90% | 1 (Reference) | 1 (Reference) | 1 (Reference) |
|  | **Yes** | 9486 | 1607, 16.94% | 6.821 (6.113-7.610) | 8.779  (7.861-9.860) | 4.833 (4.307-5.424) |
| **Validation cohort** | **No** | 216 | 13, 6.02% | 1 (Reference) | 1 (Reference) | 1 (Reference) |
|  | **Yes** | 149 | 32, 21.48% | 3.12  (1.981-7.239) | 4.710  (2.335-9.499) | 4.320  (2.074-8.997) |
| **Gastrointestinal** Tumor | **Mets** | **Total, n** | **Events, n (%)** | **Model** 1 OR (95% CI) | **Model** 2 Adjusted OR (95% CI) | **Model** 3 Adjusted **OR (95% CI)** |
| **Development cohort** | **No** | 14948 | 79, 0.53% | 1 (Reference) | 1 (Reference) | 1 (Reference) |
|  | **Yes** | 9486 | 116, 1.22% | 2.330 (1.749-3.105) | 1.465 (1.094-1.962) | 2.413 (1.780-3.217) |
| **Validation cohort** | **No** | 216 | 8, 3.70% | 1 (Reference) | 1 (Reference) | 1 (Reference) |
|  | **Yes** | 149 | 16, 10.74% | 3.128  (1.302-7.512) | 3.219  (1.237-8.376) | 4.257  (1.648-10.991) |

**Sensitivity Analysis Tables with Titles and Legends**

**Table S1. Association of Metabolic Syndrome (Defined by IDF Criteria) with Incidence of Fatty Liver Disease and Gastrointestinal Tumors**

Model 1 = unadjusted; Model 2 = adjusted for demographic and lifestyle factors (age, sex, smoking, alcohol use; additional sociodemographic variables including race, education level, and poverty-income ratio for the development cohort); Model 3 = further adjusted for laboratory parameters (white blood cell count, alanine aminotransferase, albumin, creatinine, platelet count). MetS was defined by the International Diabetes Federation (IDF) criteria, with Asian-specific waist circumference thresholds (≥90 cm for men and ≥80 cm for women) applied to the validation cohort. CI = confidence interval; OR = odds ratio; MetS = metabolic syndrome.

**Table S2. Association of Metabolic Syndrome (Defined by IDF Criteria) with Survival Outcomes in Overall Population**

| **All-cause mortality** | | | | | | |
| --- | --- | --- | --- | --- | --- | --- |
| **Overall Population** | **Mets** | **Total, n** | **Events, n (%)** | **Model 1 OR (95% CI)** | **Model 2 Adjusted HR (95% CI)** | **Model 3 Adjusted HR (95% CI)** |
| **Development cohort** | **No** | 14948 | 1361, 9.10% | 1 (Reference) | 1 (Reference) | 1 (Reference) |
|  | **Yes** | 9486 | 1585, 16.71% | 2.036  (1.894-2.190) | 1.174 (1.091-1.264) | 1.730 (1.602-1.868) |
| **Validation cohort** | **No** | 216 | 15,6.94% | 1 (Reference) | 1 (Reference) | 1 (Reference) |
|  | **Yes** | 149 | 31, 20.81% | 3.110  (1.679-5.761) | 1.770  (0.934-3.355)* | 3.071  (1.583-5.957) |
| **Cancer-specific mortality** | | | | | | |
| **Overall Population** | **Mets** | **Total, n** | **Events, n (%)** | **Model 1 OR (95% CI)** | **Model 2 Adjusted HR (95% CI)** | **Model 3 Adjusted HR (95% CI)** |
| **Development cohort** | **No** | 14948 | 336, 2.25% | 1 (Reference) | 1 (Reference) | 1 (Reference) |
|  | **Yes** | 9486 | 374, 3.94% | 1.930 (1.665-2.236) | 1.156 (0.996-1.342)* | 1.591 (1.363-1.858) |
| **Validation cohort** | **No** | 216 | 2, 0.93% | 1 (Reference) | 1 (Reference) | 1 (Reference) |
|  | **Yes** | 149 | 9, 6.04% | 5.975  (1.269-28.145) | 3.523  (0.724-17.156)* | 8.284  (1.606-42.735)* |
| **Cardiovascular mortality** | | | | | | |
| **Overall Population** | **Mets** | **Total, n** | **Events, n (%)** | **Model 1 OR (95% CI)** | **Model 2 Adjusted HR (95% CI)** | **Model 3 Adjusted HR (95% CI)** |
| **Development cohort** | **No** | 14948 | 403, 2.70% | 1 (Reference) | 1 (Reference) | 1 (Reference) |
|  | **Yes** | 9486 | 532, 5.61% | 2.319 (2.037-2.640) | 1.325 (1.163-1.510) | 2.097 (1.827-2.406) |
| **Validation cohort** | **No** | 216 | 4, 1.85% | 1 (Reference) | 1 (Reference) | 1 (Reference) |
|  | **Yes** | 149 | 8, 5.37% | 3.385  (1.042-10.996) | 2.497  (0.704-8.851)* | 4.006  (0.974-14.472)* |

Model 1 = unadjusted; Model 2 = adjusted for demographic and lifestyle factors (age, sex, smoking, alcohol use; additional sociodemographic variables including race, education level, and poverty-income ratio for the development cohort); Model 3 = further adjusted for laboratory parameters (white blood cell count, alanine aminotransferase, albumin, creatinine, platelet count). MetS was defined by the International Diabetes Federation (IDF) criteria, with Asian-specific waist circumference thresholds (≥90 cm for men and ≥80 cm for women) applied to the validation cohort. *p > 0.05, not statistically significant. CI = confidence interval; HR = hazard ratio; MetS = metabolic syndrome.
